# Numerical solution of an electrically conducting spinning flow of hybrid nanofluid comprised of silver and gold nanoparticles across two parallel surfaces

**DOI:** 10.1038/s41598-023-33520-5

**Published:** 2023-05-03

**Authors:** Aisha M. Alqahtani, Muhammad Bilal, Aatif Ali, Theyab R. Alsenani, Sayed M. Eldin

**Affiliations:** 1 Department of Mathematical Sciences, College of Science, Princess Nourah Bint Abdulrahman University, P. O. Box 84428, Riyadh, 11671 Saudi Arabia; 2 Sheikh Taimur Academic Block-II, Department of Mathematics, University of Peshawar, Peshawar, 25120 Khyber Pakhtunkhwa Pakistan; 3 Department of Mathematics, Abdul Wali Khan University Mardan, Mardan, 23200 Khyber Pakhtunkhwa Pakistan; 4 Department of Electrical Engineering, College of Engineering in Al-Kharj, Prince Sattam Bin Abdulaziz University, Al-Kharj, 11942 Saudi Arabia; 5 Faculty of Engineering, Center of Research, Future University in Egypt, New Cairo, 11835 Egypt

**Keywords:** Energy science and technology, Engineering, Mathematics and computing, Nanoscience and technology

## Abstract

The analysis of the energy transport mechanism received much attention from scientists and researchers. Conventional fluids like vegetable oils, water, ethylene glycol, and transformer oil play a vital role in numerous industrial activities. In certain industrial operations, the low heat conductivity of base fluids causes significant difficulties. This inevitably led to the advancement of critical aspects of nanotechnology. The tremendous significance of nanoscience is in improving the thermal transfer process in different heating transmitting equipment. Therefore, the MHD spinning flow of hybrid nanofluid (HNF) across two permeable surfaces is reviewed. The HNF is made of silver (Ag) and gold (Au) nanoparticles (NPs) in the ethylene glycol (EG). The modeled equations are non-dimensionalized and degraded to a set of ODEs through similarity substitution. The numerical procedure parametric continuation method (PCM) is used to estimate the 1^st^ order set of differential equations. The significances of velocity and energy curves are derived versus several physical parameters. The results are revealed through Tables and Figures. It has been determined that the radial velocity curve declines with the varying values of the stretching parameter, Reynold number, and rotation factor while improving with the influence of the suction factor. Furthermore, the energy profile enhances with the rising number of Au and Ag-NPs in the base fluid.

## Introduction

Due to the theoretical and practical uses in medical, industrial, and engineering sectors, fluid flow between parallel surfaces has several applications, such as low-cost fabrication, food processing, centrifugal filtering processes, rotating apparatus, gas–solid fluidized beds, ease of multiplexing, and miniaturization. Researchers are still exploring new and exciting aspects of flow characteristics in a rotating frame between two parallel surfaces^1–3^. The magnetized hybrid nanofluid rotating flow between two surfaces featuring entropy formation was theoretically evaluated by Khan et al.^4^. Krishna et al.^5^ described the time-varying sinusoidal pressure gradient and the slip effect on the magnetohydrodynamic (MHD) convective rotating flow. The findings show that the boundary layer thins out as the fluid motion is resisted by an elastic and magnetic field. A micropolar hybrid nanofluid’s rotational flow and heat transference within the rotatable surface were addressed by Islam et al.^6^. The findings show that increasing magnetic parameter values raises the velocity curve. The 3D spinning flow of Jeffrey fluid was addressed by Fiza et al.^7^ employing MHD and the Hall Current effect between two parallel surfaces. Heat transmission through magnetized rotational flow across an elastic sheet was addressed by Shahzad et al.^8^. Based on the findings, the rotational flow has a negative impact on heat transfer since it increases drag force. The MHD nanofluid flow was assessed by Bilal et al.^9^. A hybrid nanofluid’s rotational squeezing flow between two parallel surfaces with entropy formation was analyzed by Ali et al.^10^. The features of the hydromagnetic radiative HNF rotating flow between the two shrinking discs were investigated by Yaseen et al.^11^. The upshot of MHD on the entropy optimization of nanofluid rotational flow over a spinning disc was observed by Alqarni et al.^12^. The MHD Casson nanoliquid flow was discussed by Alqahtani et al.^13^ over an elongating surface. A permeable medium was employed by Ramzan et al.^14^ to evaluate the hydrodynamic and thermal radiation features of the rotating flow of two distinct water-based hybrid nanofluids with the variation of particle sizes. Some related studies may be found in Ref.^15–18^.

The formation of a hybrid nanofluid is accomplished by combining two distinct kinds of NPs with the base fluid. When nanoparticles are appropriately dispersed, hybrid nanofluids can provide substantial benefits in addition to their anomalously high thermal conductivity. Numerous engineering industrial sectors, including microelectronics, manufacturing, microfluidics, medicine, etc. can benefit from the application of hybrid nanofluids for heat transmission^19^. Gul et al.^20^ reported a computational study to investigate the HNF flow over an enlarging surface. Due to its effective thermophysical behavior, nanoliquid performs more effectively than basic nanofluids. Tlili et al.^21^ evaluated the magnetized HNF flow across an elongating surface with slip effects and non-uniform thickness. The results demonstrate that hybrid nanofluids are more resistant to the effects of the Lorentz force than nanofluids. Considering blood as a base fluid, Manzoor et al.^22^ calculated the energy communication through HNF with magnetic dipole characteristics over a scattering sheet. Wahid et al.^23^ analyzed the flow of a Marangoni HNF across an implanted infinitely permeable disc. Waqas et al.^24^ scrutinized the consequence of thermal radiations on HNF flow as it passed through a revolving disc. Kumar et al.^25^ described the heat transport through HNF flow along a stretchy cylinder. In a squeezed channel using engine oil as base fluid, Chu et al.^26^ reported an unsteady viscous fluid flow of gold-silver hybrid nanofluid of irregular configurations. The findings show that hybrid nanoparticles perform better than nanofluids. Forced convection in a 3D heat sink was analyzed numerically by Wang et al.^27^. The upshot of heat generation and thermal conduction on the flow of ferromagnetic HNF through a permeable substrate with slip effects were studied by Eid and Nafe^28^. It is observed from the result that increased hybrid nanoparticle concentration improves heat transfer in a shrinkable container. Alqahtani et al.^29^ estimated the impact of the slip effect and varying thickness on 3D HNF stagnation point flow along a stretchy heated curved cylinder. Tayebi et al.^30^ documented the thermal convection of an Al2O3/H2O nanoliquid contained between two rotating cylinders. Chamkha et al.^31^ used the numerical approach to analyze the MHD HNF in a closed container under the impact of the shape of nanomaterials and thermal radiation. The results showed that the local Nusselt numbers are more significantly affected by laminar nanomaterials than by other nanomaterial shapes. Seyyedi et al.^32^ used a numerical algorithm to resolve the entropy generation and heat transfer evaluations for a hexagonal cavity filled with Cu-H2O nanofluid and exposed to an aligned magnetic field. The findings showed that, for greater values of magnetic factor, the Nusselt numbers increases. Some further related studies may be found in Ref.^33–40^.

The influence of magnetism on magnetic materials, electric currents, and moving electric charges, is characterized by a vector field known as the magnetic field. Hannes Alfven first brought the concept of magnetohydrodynamics (MHD) to the world in 1970. Magnetically forced electrical conductivity in fluids is the focus of MHD. Electrolytes, plasmas, and liquid metals are all examples of materials with magnetic moments. Many different fields, including astronomy, geophysics, aviation, electromagnetic pumping, plasma jets, and MHD power generation, have found usage for MHD^41^. Alotaibi et al.^42^ calculated the impact that thermal absorption and injection have on the magnetohydrodynamic behavior of a fluid flow of Casson nanofluid in a boundary layer across a non-linear stretched surface. The 3-dimensional incompressible MHD models with magnetic diffusion and partial dissipation were analyzed by Wu and Zhu^43^. Armaghani et al.^44^ reviewed the impact of the location and size of the source and sink of heat on MHD mixed convection in the hybrid nanofluid within the L-shaped cavity. The results show that the most power applied to the sink yields the most efficient heat transmission. Patil et al.^45^ and Elayarani et al.^46^ reported the unsteady 2D nanoliquid flow containing gyrotactic micro-organisms. Vishalakshi et al.^47^ and Khashi’ie et al.^48^ evaluated the magnetized flow of HNF across a revolving plate with the Joule heating effect. According to the outcomes, an increase in the magnetic effect makes heat transmission more efficient. Abdelhameed^49^ examined how the existence of MHD and the porosity of sodium-alginate fluid affected the formation of entropy. It is noted from the result that the Bejan number effect the velocity curve. Kodi and Mopuri^50^ inspected the MHD HNF flow across a permeable substrate with a chemical reaction.

As we have discussed that the analysis of energy transport mechanism received much attention among scientists and researchers. But in certain industrial operations, the low heat conductivity of base fluids causes complications. The tremendous significance of nanoscience is in improving the thermal transfer process in different heating transmitting equipment. Therefore, the present work aims to assess the MHD spinning flow of an EG-based HNF across two permeable surfaces. The hybrid nanofluid is made of Ag-NPs, Au-NPs, and ethylene glycol. The flow mechanism is studied under the impact of a constant magnetic field. The numerical procedure PCM is used to estimate the 1^st^ order set of ODEs. The significances of velocity and energy curves are derived versus several physical parameters.

## Mathematical formulation

**Figure 1:**
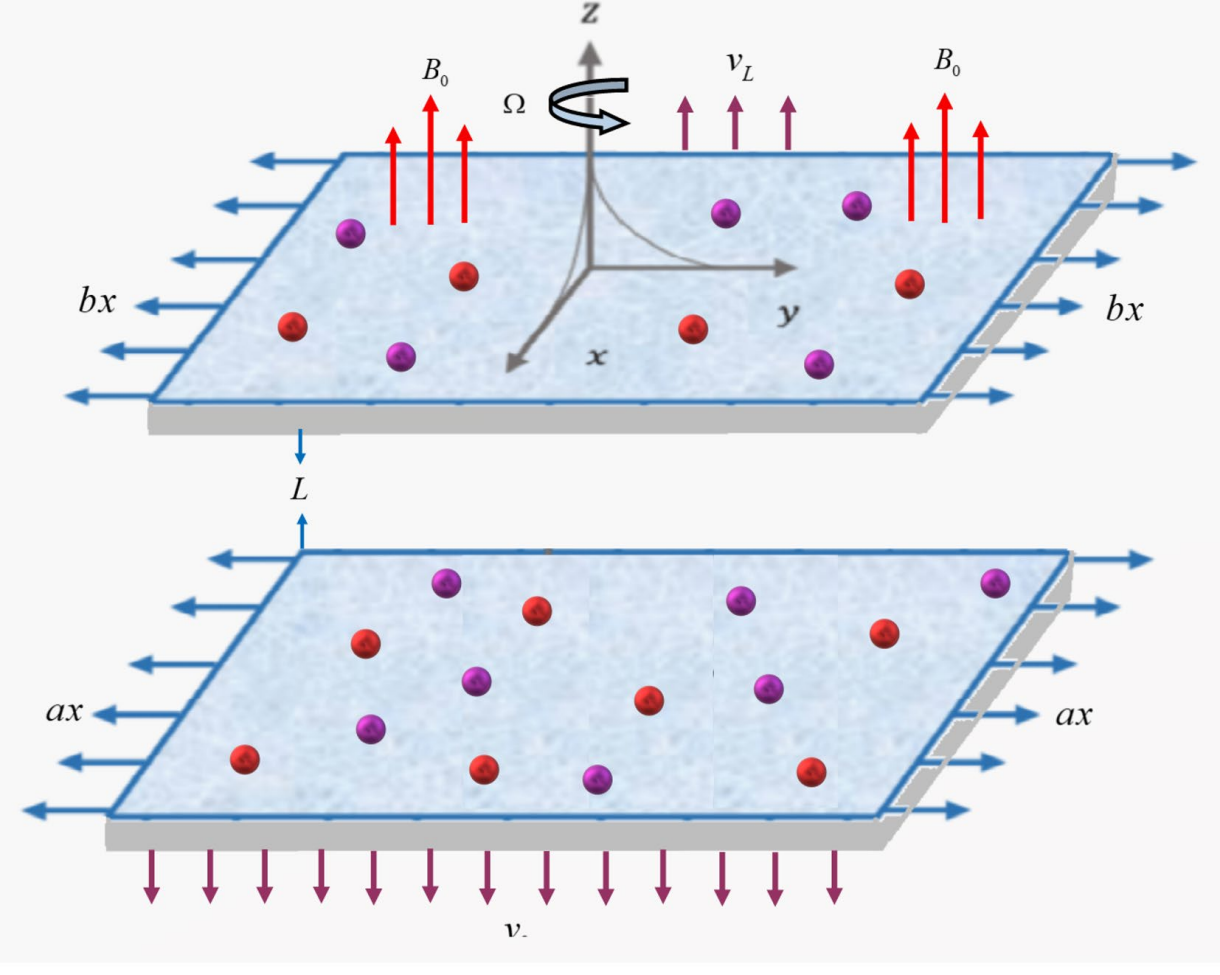
Hybrid nanofluid flow across parallel surfaces.

We have assumed steady and an incompressible HNF flow across two parallel surfaces, apart from each other at a distance of *L*. The conducting hybrid nanoliquid comprised of silver (Ag) and gold (Au) nanoparticles are perpendicularly exposed to the constant magnetic field $\left( {B_{0} } \right)$ in the *y*-direction. The parallel surfaces are considered porous and allow injection & suction. Both surfaces (upper at $y = L$ and lower at $y = 0$) are stretching with the velocity $bx$ & $ax$ as exposed in Fig. 1. The system (surfaces and fluid) rotates with an angular velocity $\left( \Omega \right)$ in the *y*-axis. The flow is mathematically expressed as^51,52^:

1 $$\frac{\partial u}{{\partial x}} + \frac{\partial v}{{\partial y}} = 0,$$

2 $$u\frac{\partial u}{{\partial x}} + v\frac{\partial u}{{\partial y}} + 2\Omega w = - \frac{1}{{\rho_{hnf} }}\frac{\partial p}{{\partial x}} + \left( {\frac{{\mu_{hnf} }}{{\rho_{hnf} }}} \right)\left( {\frac{{\partial^{2} u}}{{\partial x^{2} }} + \frac{{\partial^{2} u}}{{\partial y^{2} }}} \right) - \frac{{\sigma_{hnf} B_{0}^{2} }}{{\rho_{hnf} }}u,$$

3 $$u\frac{\partial v}{{\partial x}} + v\frac{\partial v}{{\partial y}} = - \frac{1}{{\rho_{hnf} }}\frac{\partial p}{{\partial y}} + \left( {\frac{{\mu_{hnf} }}{{\rho_{hnf} }}} \right)\left( {\frac{{\partial^{2} u}}{{\partial x^{2} }} + \frac{{\partial^{2} u}}{{\partial y^{2} }}} \right),$$

4 $$u\frac{\partial w}{{\partial x}} + v\frac{\partial w}{{\partial y}} - 2\Omega u = \left( {\frac{{\mu_{hnf} }}{{\rho_{hnf} }}} \right)\left( {\frac{{\partial^{2} w}}{{\partial x^{2} }} + \frac{{\partial^{2} w}}{{\partial y^{2} }}} \right) - \frac{{\sigma_{hnf} B_{0}^{2} }}{{\rho_{hnf} }}w,$$

5 $$u\frac{\partial T}{{\partial x}} + v\frac{\partial T}{{\partial y}} = \frac{{k_{hnf} }}{{\left( {\rho C_{{_{p} }} } \right)_{hnf} }}\left( {\frac{{\partial^{2} u}}{{\partial x^{2} }} + \frac{{\partial^{2} u}}{{\partial y^{2} }}} \right) - \frac{1}{{\left( {\rho C_{{_{p} }} } \right)_{hnf} }}\frac{{\partial q_{r} }}{\partial y} + Q_{0} \left( {T - T_{\infty } } \right).$$

The boundary conditions are:

6 $$\begin{gathered} u = ax,\,\,v = v_{0} ,\,\,w = 0,\,\,T = T_{0} \,\,\,\,\,\,\,\,\,{\text{at}}\,\,y = 0, \hfill \\ u = bx,\,\,v = v_{L} ,\,\,w = 0,\,\,T = T_{L} \,\,\,\,\,\,\,\,\,{\text{at}}\,\,y = L, \hfill \\ \end{gathered}$$

here *x* & *y* represent the rectangular coordinate, *u*, *v*, *w* are the components of velocity, $k_{hnf}$ is the thermal conductivity, $\left( {\rho c_{p} } \right)_{hnf}$ is the heat capacity. $\rho_{hnf}$ is the density and $\sigma_{hnf}$ is the electrical conductivity, $q_{r}$ is the thermal radiative heat flux, $Q_{0}$ is the heat source,$p$ is the pressure, $\left( {v_{L} > 0} \right)$ is the injection velocity at the upper surface and $v_{0} < 0$ is the suction velocity at the lower surface.

The following transformations are used:

7 $$u = axf^{\prime } \left( \eta \right),\,\,\,v = - aLf\left( \eta \right),\,\,\,w = axf\left( \eta \right),\,\,\,\theta = \frac{{T - T_{L} }}{{T_{0} - T_{L} }},\,\,\,\eta = \frac{y}{L}.$$

As a result of Eq. (7), we get:

8 $$f^{\prime \prime \prime \prime } + \left( {\frac{{\rho_{hnf} }}{{\rho_{f} }}\frac{{\mu_{f} }}{{\mu_{hnf} }}} \right)Re\left( {ff^{\prime \prime \prime } - f^{\prime } \,f^{\prime \prime } } \right) - 2R_{0} g^{\prime } \left( {\frac{{\rho_{hnf} }}{{\rho_{f} }}\frac{{\mu_{f} }}{{\mu_{hnf} }}} \right) - Mn\left( {\frac{{\sigma_{hnf} }}{{\sigma_{f} }}\frac{{\mu_{f} }}{{\mu_{hnf} }}} \right)f^{\prime \prime } g^{\prime } = 0,$$

9 $$g^{\prime \prime } + \left( {\frac{{\rho_{hnf} }}{{\rho_{f} }}\frac{{\mu_{f} }}{{\mu_{hnf} }}} \right){\text{Re}}\left( {fg^{\prime } - f^{\prime } \,g} \right) - 2R_{0} \left( {\frac{{\rho_{hnf} }}{{\rho_{f} }}\frac{{\mu_{f} }}{{\mu_{hnf} }}} \right)g^{\prime } - Mn\left( {\frac{{\mu_{f} }}{{\mu_{hnf} }}\frac{{\sigma_{hnf} }}{{\sigma_{f} }}} \right)g = 0,$$

10 $$\left( {1 + \frac{4}{3}R_{d} } \right)\frac{{k_{f} }}{{k_{hnf} }}\theta^{\prime \prime } + \frac{{\left( {\rho c_{p} } \right)_{hnf} }}{{\left( {\rho c_{p} } \right)_{f} }}\frac{{k_{f} }}{{k_{hnf} }}{\text{Re}} \Pr f\theta^{\prime} + Hs\theta = 0.$$

The reduced conditions are:

11 $$\left. {\begin{array}{*{20}l} {f^{\prime } \left( 0 \right) = 1,\,\,\,f\left( 0 \right) = \alpha ,\,\,\,\theta \left( 0 \right) = 1,\,\,\,g\left( 0 \right) = 0\,\,\,\,\,\,{\text{at}}\,\,\,\,\,\eta { = 0,}} \hfill \\ {f^{\prime } \left( 1 \right) = \lambda ,\,\,f\left( 1 \right) = \beta ,\,\,\,\theta \left( 1 \right) = 0,\,\,\,g\left( 1 \right) = 0\,\,\,\,\,\,\,{\text{at}}\,\,\,\,\,\eta { = 1}{\text{.}}} \hfill \\ \end{array} } \right\}$$

Here $\beta = - \frac{{v_{L} }}{aL}$ and $\alpha = - \frac{{v_{0} }}{aL}$ are the injection and suction factors for upper and lower surfaces, $\lambda = \frac{b}{a}$ is the stretching factor, ${\text{Re}} = \frac{{\sigma^{*} \left( {T_{2} } \right)^{3} }}{{k^{*} k_{f} }}$ is the radiation factor, ${\text{Re}} = \frac{{aL^{2} \rho_{f} }}{{\mu_{f} }}$ is the Reynolds number, ${\text{Pr}} = \frac{{Cp_{f} \mu_{f} }}{{k_{f} }}$ is the Prandtl number, $R_{0} = \frac{{L^{2} \Omega \rho_{f} }}{{\mu_{f} }}$ is the rotation constraint and $Mn = \frac{{B_{0}^{2} L^{2} \sigma_{f} }}{{\mu_{f} }}$ magnetic term.

The physical interest quantities are:

12 $$\left. {C_{f} = \frac{{ - 2\mu_{hnf} }}{{\rho_{hnf} u_{0}^{2} }}\frac{\partial u}{{\partial y}}} \right|_{y = 0} ,\,\,\,or\,\,\,C_{f} = - 2\frac{{\mu_{hnf} }}{{\mu_{f} }}\frac{{\rho_{f} }}{{\rho_{hnf} }}f^{\prime \prime } \left( 0 \right).$$

13 $${\text{Nu}} = \frac{ - L}{{k_{f} \left( {T_{0} - T_{L} } \right)}}\left( {k_{hnf} } \right)\left. {\frac{\partial T}{{\partial y}}} \right|_{y = 0} ,\,\,\,or\,\,\,{\text{Nu}} = - \frac{{k_{hnf} }}{{k_{f} }}\theta^{\prime } \left( 0 \right).$$

## Numerical solution

A details explanation related to PCM methodology is followed^54–56^:

*Step 1* Generalization to 1st order ODE

14 $$\left. {\begin{array}{*{20}l} {{\bar{\uplambda }}_{1} = f(\eta ),\,\,\,\,{\bar{\uplambda }}_{3} = f^{{\prime \prime }} (\eta ),\,\,\,\,\,{\bar{\uplambda }}_{5} = g(\eta ),\,\,\,\,{\bar{\uplambda }}_{7} = \theta (\eta ),} \hfill \\ {{\bar{\uplambda }}_{2} = f^{\prime } (\eta ),\,\,\,{\bar{\uplambda }}_{4} = f^{{\prime \prime \prime }} (\eta ),\,\,\,{\bar{\uplambda }}_{6} = \,g^{\prime } (\eta ),\,\,\,{\bar{\uplambda }}_{8} = \theta ^{\prime } (\eta ).} \hfill \\ \end{array} } \right\}$$

By putting Eq. (14) in Eqs. (8)–(10) and (11), we get:

15 $${\bar{\uplambda }}_{4}^{\prime } + \frac{{\rho _{{hnf}} }}{{\rho _{f} }}\frac{{\mu _{f} }}{{\mu _{{hnf}} }}{\text{Re}}\left( {{\bar{\uplambda }}_{1} {\bar{\uplambda }}_{4} - {\bar{\uplambda }}_{2} {\bar{\uplambda }}_{3} } \right) - 2R_{0} {\bar{\uplambda }}_{6} \frac{{\rho _{{hnf}} }}{{\rho _{f} }}\frac{{\mu _{f} }}{{\mu _{{hnf}} }} - Mn\frac{{\sigma _{{hnf}} }}{{\sigma _{f} }}\frac{{\mu _{f} }}{{\mu _{{hnf}} }}{\bar{\uplambda }}_{3} {\bar{\uplambda }}_{6} = 0,$$

16 $${\bar{\uplambda }}_{6}^{\prime } + \frac{{\rho _{{hnf}} }}{{\rho _{f} }}\frac{{\mu _{f} }}{{\mu _{{hnf}} }}{\text{Re}}\left( {{\bar{\uplambda }}_{1} {\bar{\uplambda }}_{6} - {\bar{\uplambda }}_{2} {\bar{\uplambda }}_{5} } \right) - 2R_{0} \frac{{\rho _{{hnf}} }}{{\rho _{f} }}\frac{{\mu _{f} }}{{\mu _{{hnf}} }}{\bar{\uplambda }}_{6} - Mn\frac{{\mu _{f} }}{{\mu _{{hnf}} }}\frac{{\sigma _{{hnf}} }}{{\sigma _{f} }}{\bar{\uplambda }}_{5} = 0,$$

17 $$\left( {1 + \frac{4}{3}R_{d} } \right)\frac{{k_{f} }}{{k_{hnf} }}{{\bar{\uplambda}}}_{8}^{\prime } + \frac{{\left( {\rho c_{p} } \right)_{hnf} }}{{\left( {\rho c_{p} } \right)_{f} }}\frac{{k_{f} }}{{k_{hnf} }}{\text{Re}}\;{\text{Pr}}\;{{\bar{\uplambda}}}_{1} {{\bar{\uplambda}}}_{8} + Hs{{\bar{\uplambda}}}_{7} = 0.$$

The transform conditions are:

18 $$\begin{gathered} {{\bar{\uplambda}}}_{1} \left( 0 \right) = \alpha ,\,\,\,{{\bar{\uplambda}}}_{2} \left( 0 \right) = 1,\,\,\,{{\bar{\uplambda}}}_{5} \left( 0 \right) = 0,\,\,\,{{\bar{\uplambda}}}_{7} \left( 0 \right) = 1\quad {\text{at}}\quad \eta { = 0,} \hfill \\ {{\bar{\uplambda}}}_{1} \left( 1 \right) = \beta ,\,\,\,{{\bar{\uplambda}}}_{2} \left( 1 \right) = \lambda ,\,\,\,{{\bar{\uplambda}}}_{5} \left( 1 \right) = 0,\,\,\,{{\bar{\uplambda}}}_{7} \left( 1 \right) = 0\quad {\text{at}}\quad \eta { = 1}{\text{.}} \hfill \\ \end{gathered}$$

*Step 2* Introducing parameter *p* in Eq. (16)–(20):

19 $${\bar{\uplambda }}_{4}^{\prime } + \frac{{\rho _{{hnf}} }}{{\rho _{f} }}\frac{{\mu _{f} }}{{\mu _{{hnf}} }}{\text{Re}}\left( {{\bar{\uplambda }}_{1} {\bar{\uplambda }}_{4} - {\bar{\uplambda }}_{2} {\bar{\uplambda }}_{3} } \right) - 2R_{0} {\bar{\uplambda }}_{6} \frac{{\rho _{{hnf}} }}{{\rho _{f} }}\frac{{\mu _{f} }}{{\mu _{{hnf}} }} - Mn\frac{{\sigma _{{hnf}} }}{{\sigma _{f} }}\frac{{\mu _{f} }}{{\mu _{{hnf}} }}{\bar{\uplambda }}_{3} {\bar{\uplambda }}_{6} = 0,$$

20 $${\bar{\uplambda }}_{6}^{\prime } + \frac{{\rho _{{hnf}} }}{{\rho _{f} }}\frac{{\mu _{f} }}{{\mu _{{hnf}} }}{\text{Re}}\left( {{\bar{\uplambda }}_{1} {\bar{\uplambda }}_{6} - {\bar{\uplambda }}_{2} {\bar{\uplambda }}_{5} } \right) - 2R_{0} \frac{{\rho _{{hnf}} }}{{\rho _{f} }}\frac{{\mu _{f} }}{{\mu _{{hnf}} }}{\bar{\uplambda }}_{6} - Mn\frac{{\mu _{f} }}{{\mu _{{hnf}} }}\frac{{\sigma _{{hnf}} }}{{\sigma _{f} }}{\bar{\uplambda }}_{5} = 0,$$

21 $$\left( {1 + \frac{4}{3}R_{d} } \right)\frac{{k_{f} }}{{k_{hnf} }}{{\bar{\uplambda}}}_{8}^{\prime } + \frac{{\left( {\rho c_{p} } \right)_{hnf} }}{{\left( {\rho c_{p} } \right)_{f} }}\frac{{k_{f} }}{{k_{hnf} }}{\text{RePr}}\;\;{{\bar{\uplambda}}}_{1} {{\bar{\uplambda}}}_{8} + Hs{{\bar{\uplambda}}}_{7} = 0.$$

## Results and discussion

This segment expresses the physical mechanisms and reason behind the increasing and decreasing effect of velocity, mass, and energy outlines versus physical interest quantities. The following are some different profiles:

### Velocity interpretation

**Figure 2:**
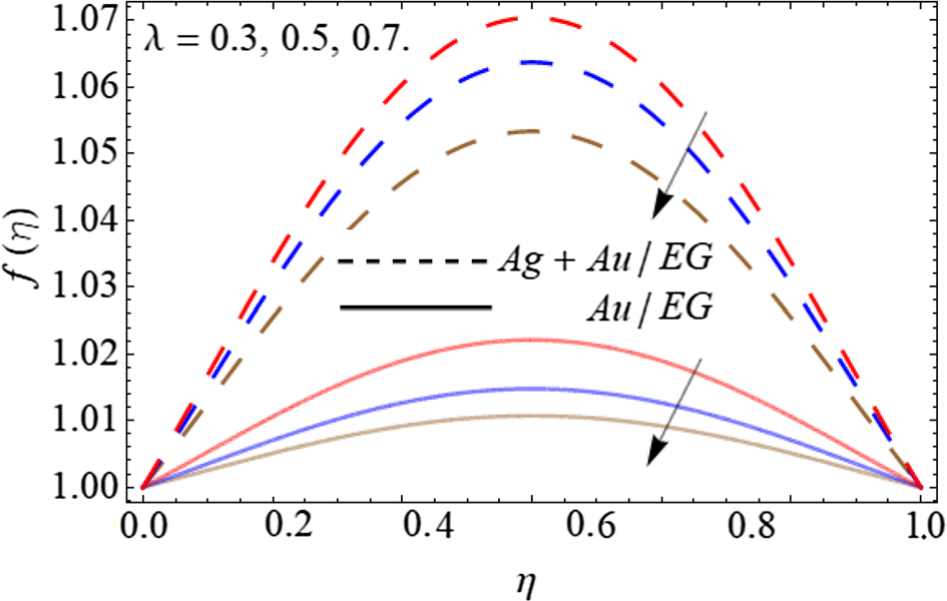
Consequence of the Stretching parameter $\lambda$ on $f\left( \eta \right)$.

**Figure 3:**
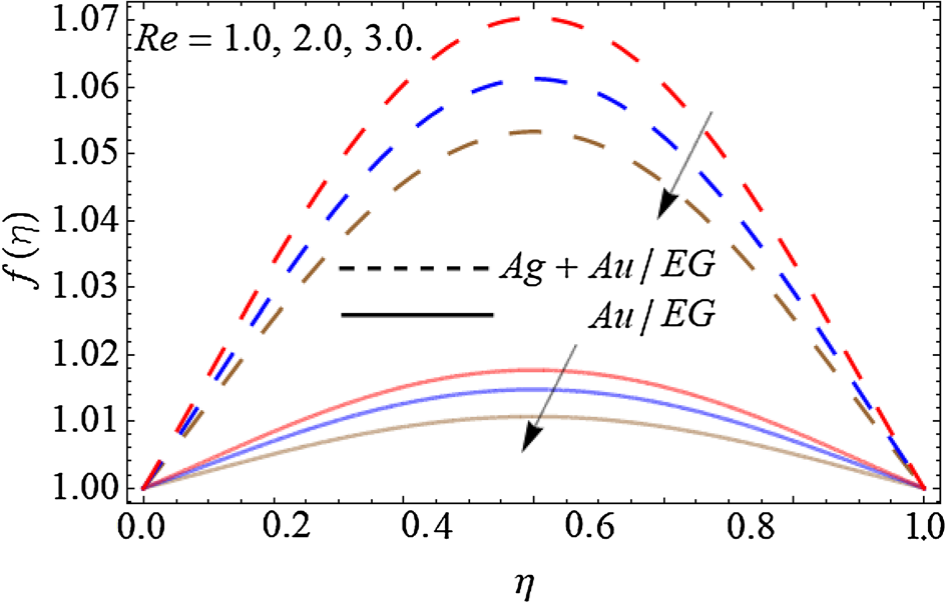
Consequence of the Reynold number $Re$ on $f\left( \eta \right)$.

**Figure 4:**
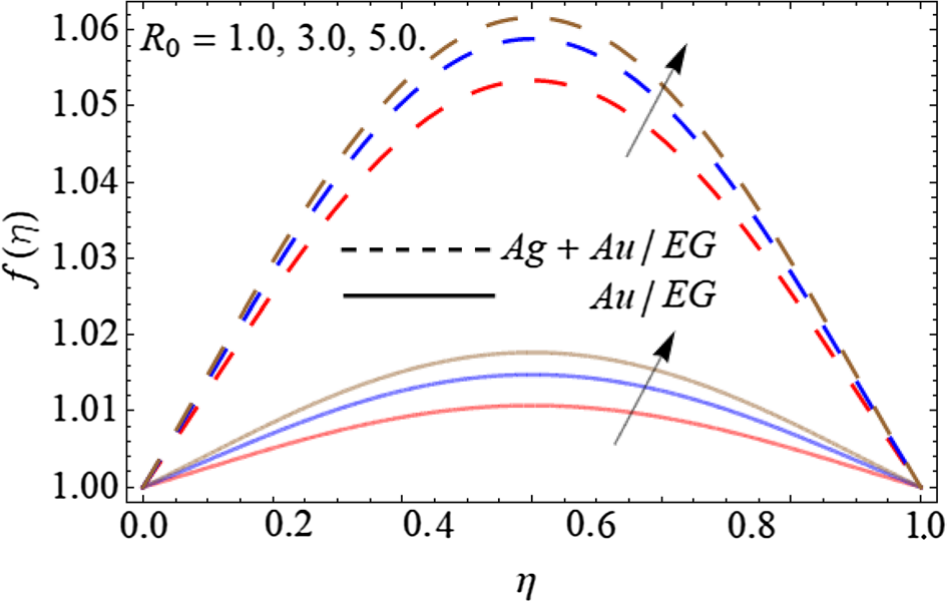
Consequence of the Rotation factor $R_{0}$ on $f\left( \eta \right)$.

**Figure 5:**
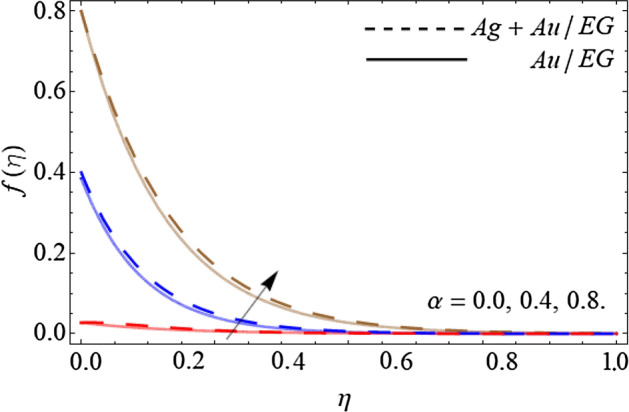
Consequence of the Suction term $\alpha$ on $f\left( \eta \right)$.

Figures 2, 3, 4 and 5 describe the effect of the stretching parameter $\lambda$, Reynold number Re, rotation factor $R_{0}$, and suction parameter $\alpha$ on the radial velocity curve $f\left( \eta \right)$. Figures 2 and 3 show that the velocity field lessens with the varying values of stretching constraint $\lambda$ and Reynold number Re. Physically, the constant stretching of the sheet generates disturbance, which resists the fluid motion and as a result drops the velocity curve $f\left( \eta \right)$ as revealed in Fig. 2. Similarly, from the mathematical expression of Reynold number ${\text{Re}} = \frac{{aL^{2} \rho_{f} }}{{\mu_{f} }}$, one can observe that the density and distance (*L*) between two surfaces increases with the effect of Reynold number, which causes the reduction of velocity curve as presented in Fig. 3. Because the flow motion boots in the narrow tube or channel as compared to wide according to the Bernoulli statement. Figures 4 and 5 exhibit that the $f\left( \eta \right)$ deteriorates with the varying values of rotation factor $R_{0}$ and suction parameter $\alpha$. Physically, the angular motion of surfaces, rotates the fluid particles adjacent to the surface according to the no-slip conditions and as a result, the velocity outline enhances as exposed in Fig. 4. Figure 5 expresses that the $f\left( \eta \right)$ develops with the upshot of suction factor.

**Figure 6:**
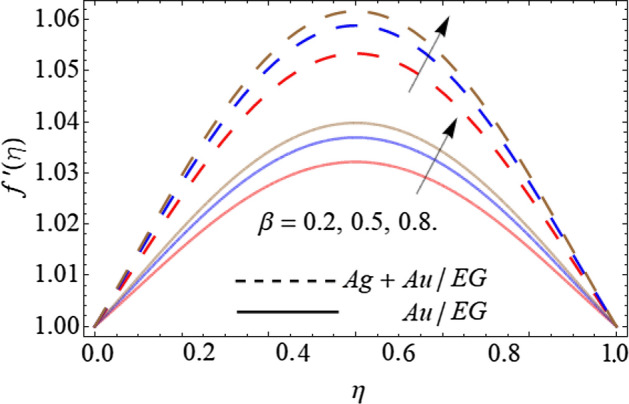
Consequence of the Injection parameter $\beta$ on $f^{\prime } \left( \eta \right)$.

**Figure 7:**
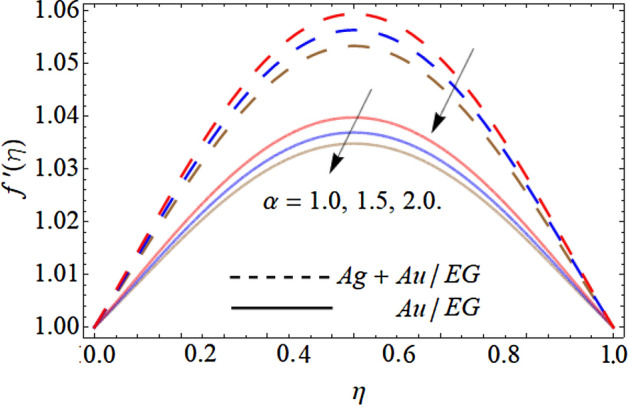
Consequence of the Suction parameter $\alpha$ on $f^{\prime } \left( \eta \right)$.

**Figure 8:**
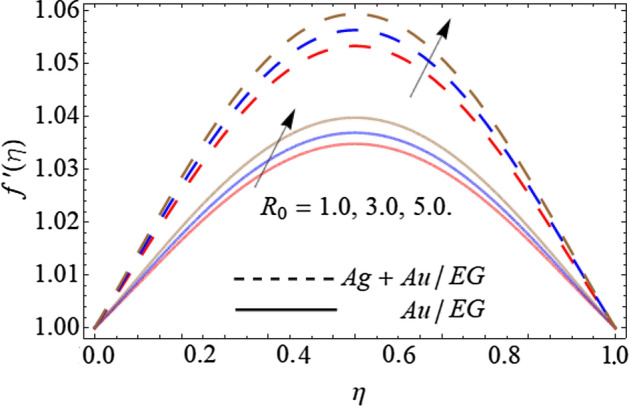
Consequence of the Rotation parameter $\beta$ on $f^{\prime } \left( \eta \right)$.

**Figure 9:**
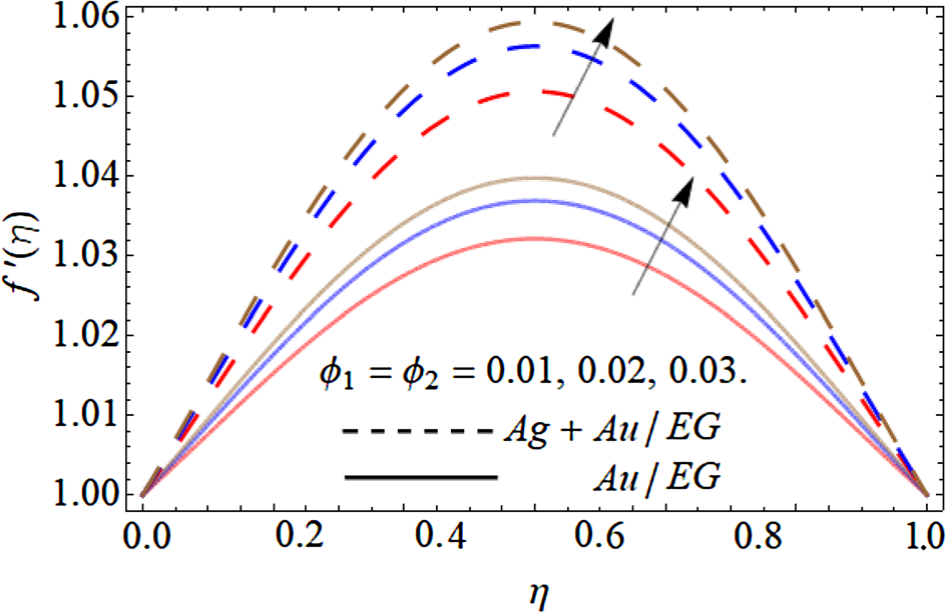
Consequence of the rising numbers of Nanoparticles on $f^{\prime } \left( \eta \right)$.

Figures 6, 7, 8 and 9 describe the effect injection parameter $\beta$, suction parameter $\alpha$, rotation parameter $\beta$, and nanoparticles volume friction $\left( {\phi_{1} ,\,\,\phi_{2} } \right)$ on the axial velocity curve $f^{\prime } \left( \eta \right)$ respectively. Figures 6 and 7 reveal that the influence of the injection parameter $\beta$ enhances, while the effect of the suction factor $\alpha$ drops the axial velocity curve $f^{\prime } \left( \eta \right)$. Figures 8 and 9 expose that the growing values of the rotation term $\beta$ and mounting numbers of nanoparticles $\left( {\phi_{1} ,\,\,\phi_{2} } \right)$ boost the velocity curve. As we have discoursed before that the angular motion of surfaces, rotates the fluid particles adjacent to the surface according to the no-slip conditions, hence, the velocity outline $f^{\prime } \left( \eta \right)$ augments as publicized in Fig. 8. On the other hand, the specific heat capacity of ethylene glycol $\left( {2430\,c_{p} \;\left( {{\text{J}}\;{\text{Kg}}^{ - 1} \;{\text{K}}^{ - 1} } \right)} \right)$ is greater than the Au and Ag NPs. Therefore, the inclusion of Au and Ag NPs in EG reduces the heat-absorbing capacity of HNF, as a result, the fluid loses its viscosity and accelerates the fluid motion $f^{\prime } \left( \eta \right)$ as revealed in Fig. 9.

### Temperature interpretation

**Figure 10:**
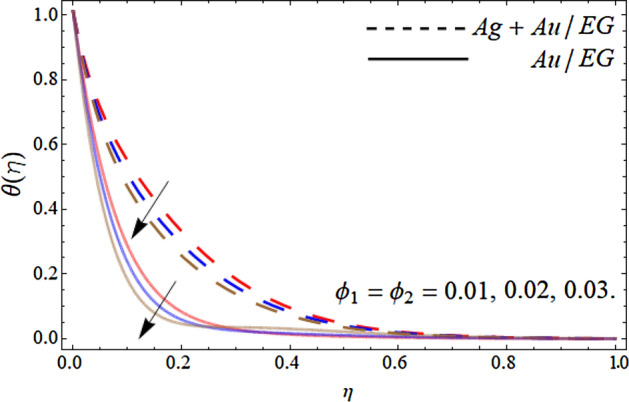
Consequence of the rising numbers of Nanoparticles on the energy curve $\theta \left( \eta \right)$.

**Figure 11:**
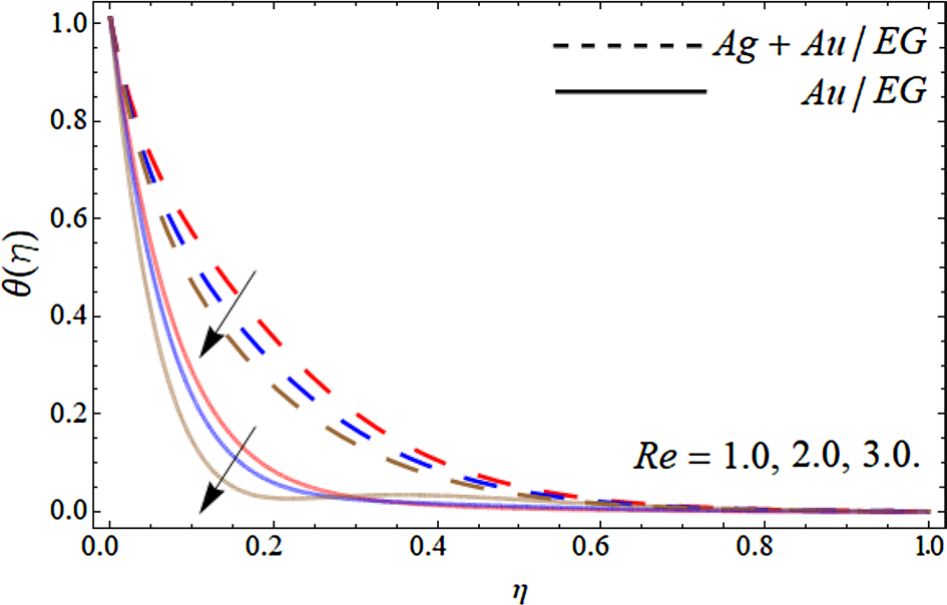
Consequence of the Reynold number *Re* on the energy curve $\theta \left( \eta \right)$.

**Figure 12:**
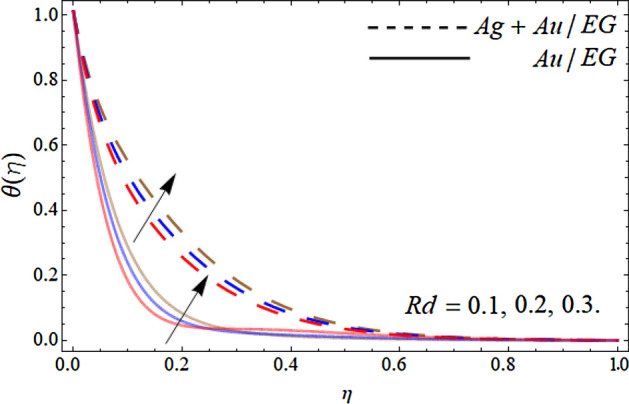
Consequence of the Radiation term on the energy curve $\theta \left( \eta \right)$.

**Figure 13:**
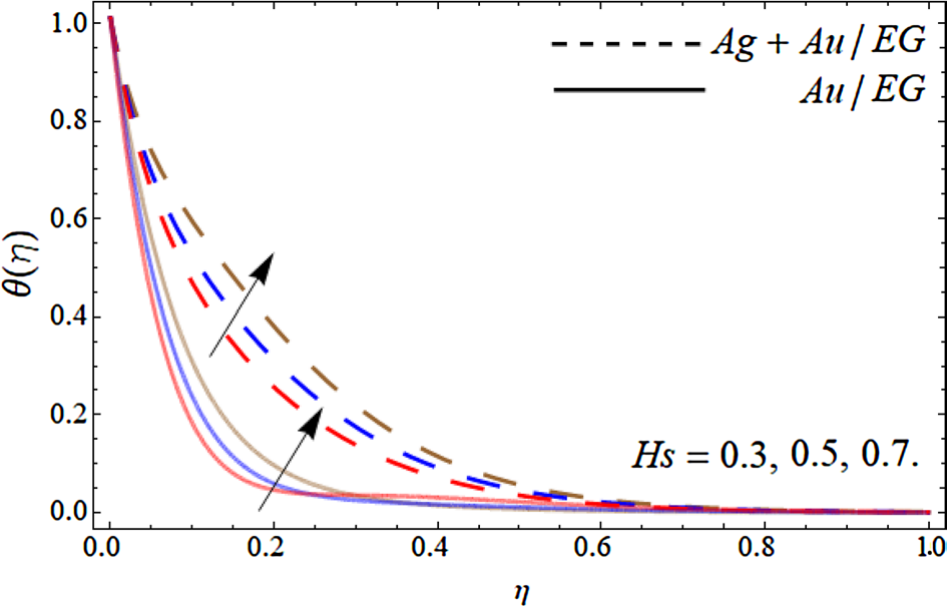
Consequence of the heat source on the energy curve $\theta \left( \eta \right)$.

Figures 10, 11, 12 and 13 pronounce the effect of nanoparticles volume friction $\left( {\phi_{1} ,\,\,\phi_{2} } \right)$, Reynold number Re, radiation term *Rd* and heat source *Hs* on the energy curve $\theta \left( \eta \right)$ respectively. Figure 10 expresses that the energy outline enhances with the rising number of Au and Ag NPs. Physically, the thermal conductivity of Ag and Au Nps is greater than EG, therefore, the addition of these NPs to EG, boosts the average thermal conductivity of hybrid nanoliquid as displayed in Fig. 10. Figure 11 shows that the varying influence of Reynold number diminishes the energy curve. Figures 12 and 13 illustrates that the energy curve $\theta \left( \eta \right)$ augments with the effect of thermal radiation term and heat source *Hs.* Physically, thermal radiation is the radiation produced by the thermal transfer of matter particulate. Thermal radiation is produced when the heat generated by charge mobility in content is transformed into electromagnetic radiation. This electromagnetic radiation, when applied to the fluid flow, accelerates the energy curve $\theta \left( \eta \right)$ as demonstrated in Fig. 12. Similarly, the heat source also enhances the energy outlines of the hybrid nanoliquid as presented in Fig. 13. Physically, the heat source/sink is a passive energy transport that communicates the energy fashioned by mechanical or an electronic apparatus into a coolant fluid in motion. The heat source is a source that radiates or generates heat.

Tables 1 and 2 elaborate the tentative values of Au-NPs and Ag-NPs and EG used for the simulation of the problem and mathematical model comprising different thermal features of the EG and nanoparticles. Table 3 expresses the comparative study of the present work against the published literature for validity purposes as well as revealed the statistical results for $C_{f}$ and Nu. It can be perceived that both physical interest quantities $\left( {C_{f} ,\,\,{\text{Nu}}} \right)$ transfer rate boost with the influence of Reynold number and NPs volume friction.

**Table 1:** The thermo-physical properties of nano particulates with base fluid^53^.

Thermo-physical properties	Base fluid	Nanoparticles	
	${\text{C}}_{2} {\text{H}}_{6} {\text{O}}_{2}$(Ethylene–Glycol)	Ag (Silver)	Au (Gold)
$\rho \;\left( {{\text{Kg}}\;{\text{m}}^{ - 3} } \right)$	1115	10,500	19,300
$c_{p} \;\left( {{\text{J}}\;{\text{Kg}}^{ - 1} \;{\text{K}}^{ - 1} } \right))$	2430	235	129.1
$k\;\left( {{\text{W}}\;{\text{m}}^{ - 1} \;{\text{K}}^{ - 1} } \right)$	0.253	429	318
$\beta \times 10^{ - 5} \;\left( {{\text{K}}^{ - 1} } \right)$	5.7	1.89	1.4
$\sigma \;\left( {{\text{S}}\;{\text{m}}^{ - 1} } \right)$	10.7 × 10^−5^	6.30 × 10^7^	4.25 × 10^7^

**Table 2:** The thermos-physical characteristics of HNF^53^.

	Nanofluids	Hybrid-nanofluid $\phi_{hnf} = (\phi_{\text{Ag}} + \phi_{\text{Au}} )$
Viscosity $\mu$	$\frac{{\mu_{f} }}{{(1 - \phi_{\text{Au}} )}}$ and $\frac{{\mu_{f} }}{{(1 - \phi_{\text{Ag}} )}}$	$\frac{{\mu_{f} }}{{(1 - \phi_{hnf} )^{2.5} }}$
Density $\rho$	$(1 - \phi_{\text{Au}} )_{\rho f} + \phi_{\text{Au}} \rho_{\text{Au}}$ and $(1 - \phi_{\text{Ag}} )_{\rho f} + \phi_{\text{Ag}} \rho_{\text{Ag}}$	$(1 - \phi_{hnf} )\rho_{f} + \phi_{\text{Ag}} \rho_{\text{Ag}} + \phi_{\text{Au}} \rho_{\text{Au}}$
Heat capacity $(\rho C_{p} )$	$(1 - \phi_{\text{Au}} )(\rho C_{p} )_{f} + \phi_{\text{Au}} (\rho C_{p} )_{\text{Au}}$ and $(1 - \phi_{\text{Ag}} )(\rho C_{p} )_{f} + \phi_{\text{Ag}} (\rho C_{p} )_{\text{Ag}}$	$(1 - \phi_{hnf} )(\rho C_{p} )_{f} + \phi_{\text{Ag}} (\rho C_{p} )_{\text{Ag}} + \phi_{\text{Au}} (\rho C_{p} )_{\text{Au}}$
Thermal conductivity $k$	$\frac{{k_{\text{Au}} + 2k_{f} - 2\phi_{\text{Au}} (k_{f} - k_{\text{Au}} )}}{{k_{\text{Au}} + 2k_{f} + 2\phi_{\text{Au}} (k_{f} - k_{\text{Au}} )}}k_{f}$ and $\frac{{k_{\text{Ag}} + 2k_{f} - 2\phi_{\text{Ag}} (k_{f} - k_{\text{Ag}} )}}{{k_{\text{Ag}} + 2k_{f} + 2\phi_{\text{Ag}} (k_{f} - k_{\text{Ag}} )}}k_{f}$	$$\begin{aligned} & \frac{{k_{\text{Au}} + 2k_{nf} - 2\phi_{\text{Au}} (k_{nf} - k_{\text{Au}} )}}{{k_{\text{Au}} + 2k_{nf} + 2\phi_{\text{Au}} (k_{nf} - k_{\text{Au}} )}} \\ & \quad \times \frac{{k_{\text{Ag}} + 2k_{f} - 2\phi_{\text{Ag}} (k_{f} - k_{\text{Ag}} )}}{{k_{\text{Ag}} + 2k_{f} + 2\phi_{\text{Ag}} (k_{f} - k_{\text{Ag}} )}}k_{f} \\ \end{aligned}$$
Diffusivity $\alpha$	$\frac{{k_{nf} }}{{(\rho C_{p} )_{nf} }}$	$\frac{{k_{hnf} }}{{(\rho C_{p} )_{hnf} }}$

**Table 3:** The statistical outcomes of skin friction and Nusselt number.

Parameters			Hafeez et al.^51^	Present work	Parameters			Hafeez et al.^51^	Present work
${\text{Re}}$	$R_{0}$	*Mn*	$C_{f}$	$C_{f}$	${\text{Re}}$	$\phi_{1} = \phi_{2}$	Pr	${\text{Nu}}$	${\text{Nu}}$
1.0			71.9017	71.90181	1.0			14.0721	14.07223
2.0			74.0635	74.06362	2.0			15.1956	15.19572
**3.0**			**76.2852**	**76.28545**	**3.0**			**16.3696**	**16.36975**
	1.5		71.9054	71.90553		3.0		6.59670	6.596728
	2.0		71.9106	71.91076		4.0		14.0721	14.07238
	**2.5**		**71.9173**	**71.91751**		**5.0**		**38.2791**	**38.27936**
		2.5	72.0311	72.03138			2.5	4.43466	4.434689
		3.0	72.1601	72.16038			3.0	4.76439	4.764473
		**3.5**	**72.2890**	**72.28923**			**3.5**	**5.10782**	**5.107858**

Significant values are in [bold].

## Conclusions

The analysis of energy and mass transport mechanism through the MHD spinning flow of an ethylene glycol based HNF across two permeable surfaces is reviewed. The hybrid nanofluid is made of Ag and Au-NPs in ethylene glycol. The solution obtained from the PCM is presented through Tables and Figures. The key conclusions are:

- The radial velocity curve declines with the varying values of the stretching parameter $\lambda$ and Reynold number Re.
- The fluid velocity curve $f\left( \eta \right)$ drops with the variation of rotation factor $R_{0}$ and suction parameter while improving with the influence of the suction factor.
- The influence of the injection parameter $\beta$ enhances, while the effect of the suction parameter $\alpha$ drops the axial velocity curve $f^{\prime } \left( \eta \right)$.
- The rising values of rotation constraint $\beta$ and mounting numbers of nanoparticles $\left( {\phi_{1} ,\,\,\phi_{2} } \right)$ boost the velocity curve.
- The energy framework enhances with the effect of thermal radiation term and heat source*.* The energy framework also enriches with the rising number of Au and Ag NPs in the base fluid.
- Both physical interest quantities $\left( {C_{f} ,\,\,{\text{Nu}}} \right)$ transfer rate boost with the influence of Reynold number and NPs volume friction.

## Data Availability

All data used in this manuscript have been presented within the article.

## List of symbols

Au: Silver
$$B_{0}$$: Magnetic field
$$\Omega$$: Angular velocity
*NPs*: Nanoparticles
$$T$$: Temperature
$$\left( {\rho c_{p} } \right)_{hnf}$$: Heat capacity
$$\left( {v_{L} > 0} \right)$$: Injection velocity
$$\mu_{hnf}$$: Dynamic viscosity
$$\lambda$$: Stretching factor
$q_{r}$: Thermal radiative heat flux
$$\beta$$: Injection parameter
Re: Reynold number
$$R_{0}$$: Rotation constraint
${\text{C}}_{2} {\text{H}}_{6} {\text{O}}_{2}$: Ethylene glycol formula
$$f\left( \eta \right)$$: Velocity profile
$$\phi_{\text{Ag}}$$: Silver nanoparticles
Ag: Gold
*L*: Distance between surfaces
*u*, *v*, *w*: Components of velocity
$$k_{hnf}$$: Thermal conductivity
$$Q_{0}$$: Heat source
$$p$$: Pressure
$$\rho_{hnf}$$: Density
$$\alpha$$: Suction parameter
$$\sigma_{hnf}$$: Electrical conductivity
$$v_{0} < 0$$: Suction velocity
*Rd*: Radiation factor
Pr: Prandtl number
*Mn*: Magnetic term
EG: Ethylene glycol symbol
$$\theta \left( \eta \right)$$: Energy profile
$$\phi_{\text{Au}}$$: Gold nanoparticles
